# A Tunable Dual-Band and Polarization-Insensitive Coherent Perfect Absorber Based on Double-Layers Graphene Hybrid Waveguide

**DOI:** 10.1186/s11671-019-3155-z

**Published:** 2019-11-04

**Authors:** Xin Luo, Zi-Qiang Cheng, Xiang Zhai, Zhi-Min Liu, Si-Qi Li, Jian-Ping Liu, Ling-Ling Wang, Qi Lin, Yan-Hong Zhou

**Affiliations:** 1 Department of Applied Physics, School of Science, East China Jiaotong University, Nanchang, 330013 People’s Republic of China; 2 Key Laboratory for Micro-Nano Optoelectronic Devices of Ministry of Education, School of Physics and Electronics, Hunan University, Changsha, 410082 China; 3 School of Electrical and Electronic Engineering, Huazhong University of Science and Technology, Hubei, 430074 People’s Republic of China; 4 College of Physics Science and Engineering Technology, Yichun University, Yichun, 336000 Jiangxi Province China

**Keywords:** Graphene, Absorption, Surface plasmons

## Abstract

A suspended monolayer graphene has only about 2.3% absorption rate in visible and infrared band, which limits its optoelectronic applications. To significantly increase graphene’s absorption efficiency, a tunable dual-band and polarization-insensitive coherent perfect absorber (CPA) is proposed in the mid-infrared regime, which contains the silicon array coupled in double-layers graphene waveguide. Based on the FDTD methods, dual-band perfect absorption peaks are achieved in 9611 nm and 9924 nm, respectively. Moreover, due to its center symmetric feature, the proposed absorber also demonstrates polarization-insensitive. Meanwhile, the coherent absorption peaks can be all-optically modulated by altering the relative phase between two reverse incident lights. Furthermore, by manipulating the Fermi energies of two graphene layers, two coherent absorption peaks can move over a wide spectrum range, and our designed CPA can also be changed from dual-band CPA to narrowband CPA. Thus, our results can find some potential applications in the field of developing nanophotonic devices with excellent performance working at the mid-infrared regime.

## Introduction

As a crucial issue for nanophotonics and optoelectronics, efficient light-matter interaction has widely caused concerns in recent years [1, 2], particularly in the atomically thin two-dimension (2D) materials. Many reports have been demonstrated, such as transition-metal dichalcogenides (TMDCs) [3, 4], graphene [5–9], hexagonal boron nitride [10], black phosphorus [11], and so on. As a prototypical 2D material, graphene can interact with light in a wide (ultraviolet to terahertz) wavelength range. However, due to its natural gapless and conical electronic band structure [12], the absorption efficiency of light in graphene is as low as about 2.3%. Fortunately, the optical bandgap of graphene can be opened up by doping or using the other special methods, which results in the excitation of surface plasmon polaritons (SPPs) in the terahertz and infrared bands [13]. Then, the absorption and confinement of light in graphene can be remarkably strengthened because of the excited SPPs, which can prolong the interaction time between graphene and light [14–19]. Therefore, graphene plasmonic devices have become an interesting and significant topic, and extensive researches have been demonstrated in various fields, such as absorbers [17, 18], optical filters [20], sensors [21], modulators [22], and photodetectors [23, 24].

More specifically, among these devices based on graphene, optical absorber takes an important role in the field of developing advanced optoelectronic devices, such as solar energy-trapping devices and emitters. Recently, due to the unique attributes of graphene, some absorbers based on graphene have been reported. Moreover, as mentioned above, most of these absorbers are focused on the terahertz and infrared regimes, because graphene with special processes can excite SPPs, leading to the strong light-graphene interactions in these wavelengths [3]. For instance, based on graphene, Luo et al. [25] proposed a tunable perfect absorber with ultra-narrowband, which can maintain satisfactory performances under broad-angle incidence. In Ref. [16], by embedding monolayer graphene to the metamaterials, Xiao et al. demonstrated that the EIT analog was realized in the terahertz regime, and its resonance intensity could be flexibly manipulated over a wide range. Jiang et al. [26] designed, fabricated, and investigated a broadband absorber based on patterned graphene in the terahertz regime, and the absorption above 90% is achieved from 1.54 to 2.23 THz. In order to manipulate the surface plasmon of graphene in an effective and feasible way, Xia et al. suggested that it could be realized by using a conductive sinusoidal grating with sub-wavelength size [19].

Importantly, coherent perfect absorber (CPA), which is another way to control and strengthen the optical absorption of graphene, has attracted great attention due to the all-optical modulation features [27, 28]. Depending on the interference effects and interplay of absorption, CPA provides a potential method to manipulate light with light without nonlinearity. Y. D. Chong et al. theoretically investigated the CPA with the scattering matrix [29]. Before long, two kinds of CPA were successively reported in the silicon slab [30] and planar metamaterial [31]. Recently, CPA has also been intensively studied in graphene-based devices. For example, combined with centrosymmetry metal-graphene nanostructure, Y. Ning et al. [32] investigated a tunable polarization-insensitive CPA and showed that the absorption could be flexibly and all-optically modulated by the Fermi energy of graphene and the relative phase between the incident lights. By trapping the guided-mode resonance in a subwavelength dielectric grating, X. Feng et al. [33] realized a tunable graphene-based CPA, which can be applied in a wide spectrum coverage from visible to infrared regimes. Y. C. Fan et al. [34] exploited graphene nanoribbon-based metasurface to CPA in the mid-infrared regime, and demonstrated that this CPA can be manipulated flexibly by changing the properties of graphene and structural parameters of the metasurface. However, the dual-band graphene-based CPA is also of great significance to the nanophotonics and optoelectronics devices, but seldom investigated in the mid-infrared regime. Furthermore, how to improve its adjustability is also a challenge facing the dual-band CPA.

In this paper, we design and study a tunable dual-band and polarization-insensitive CPA in the mid-infrared band, which contains a silicon array coupled in double-layers graphene waveguide. The physical mechanism of the designed CPA is analyzed by the scattering matrix. Meanwhile, the features of proposed CPA are demonstrated by the finite-difference time-domain (FDTD) simulations. When the incident light is illuminated into the silicon array, since the plasmonic resonances on the double continuous graphene films can be emerged due to the mechanism of guided-mode resonance, then the coupling effect between them results in the perfect dual-band absorption peaks, which are achieved in 9611 nm and 9924 nm, respectively. Moreover, due to its center symmetric feature, the proposed absorber also demonstrates polarization-insensitive. Furthermore, most of the reported graphene-based absorbers are manipulated by only changing the properties of graphene through an electrostatic field, magnetic field, or chemical doping, which are the causes of additional losses and also make the devices more complicated. For our proposed CPA, the coherent absorptions can be all-optically modulated by altering the relative phase between two reverse incident lights, which improves the absorber’s adjustability and does not increase the complexity of the structure. Meanwhile, by manipulating the Fermi energies of two graphene layers, two coherent absorption peaks can move over a wide spectrum range, and our designed CPA can also be changed from dual-band CPA to narrowband CPA. Therefore, our work provides a very promising way with convenience and sensitivity for potential applications include switches, all-optical logical devices, and coherent photodetectors.

## Methods

**Fig. 1:**
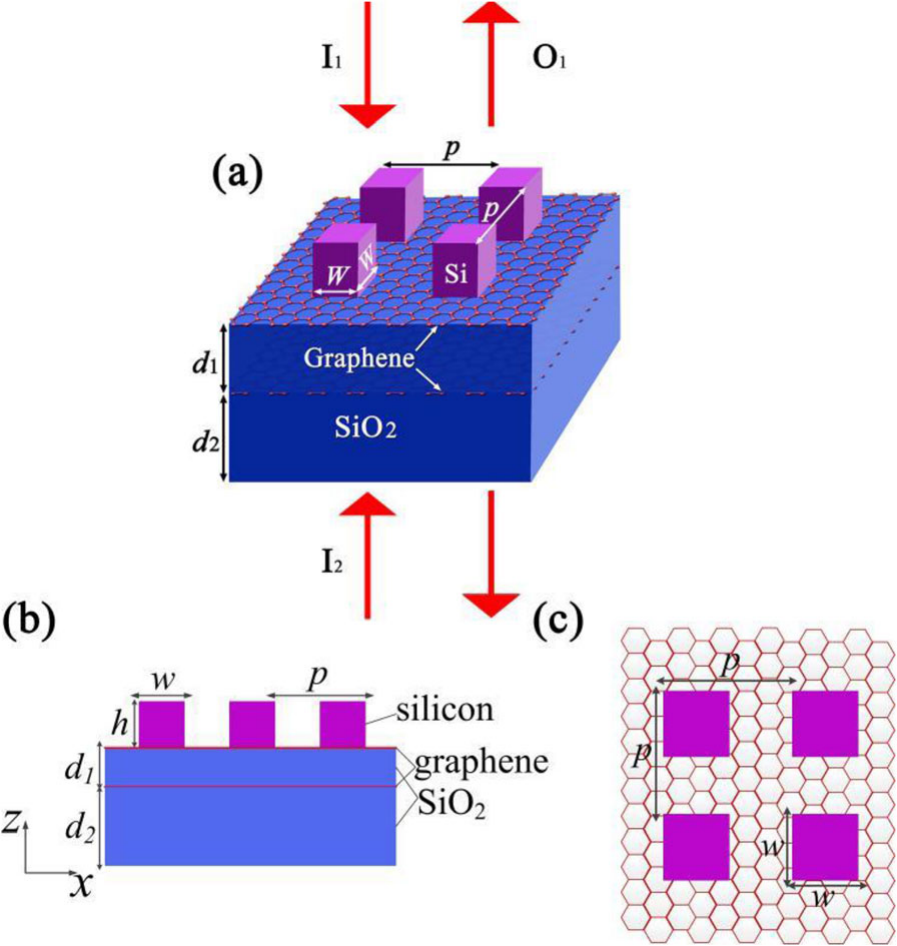
**a** Schematic diagram of the dual-band graphene-based perfect absorber. **b** Side view with dimensions specified. **c** Top view with dimensions specified

As illustrated in Fig. 1, there are two continuous graphene films on the silica substrate, which are separated by a silica layer. Meanwhile, the silicon array is put on the top of upper graphene film. Here, the length (*x*-direction) and width (*y*-direction) of every silicon square in the array are both set as *w* = 80 nm, as shown in Fig. 1c. Meanwhile, both the periods of silicon squares in the *x*-direction and *y*-direction are *p* = 160 nm, and the thickness (*z* direction) of silicon square is *h* = 100 nm. Moreover, the thicknesses of the silica spacer and substrate are *d*_1_ = 75 nm and *d*_2_ = 150 nm, respectively. *I*_*1*_ and *I*_*2*_, as two coherent incident lights, are simultaneously irradiated on the proposed CPA from two contrary directions, as shown in Fig. 1a. The relationship between *I*_*1*_ and *I*_*2*_ is *I*_2_ = *αI*_1_ exp(*iφ* + *ikz*), where *α*, *φ*, and *z* are the relative amplitude, phase difference, and phase reference point between *I*_*1*_ and *I*_*2*_, respectively. *O*_*1*_ and *O*_*2*_ are the emergent lights scattering from the bottom and top of proposed CPA. Furthermore, the thicknesses of two graphene films are both set as 0.34 nm in our simulations, and the conductivities of two graphene films are both computed within the local random phase approximation as follows [35]:

1 $$ \sigma \left(\omega \right)=\frac{ie^2{\kappa}_BT}{\pi {\mathrm{\hslash}}^2\left(\omega +i{\tau}^{-1}\right)}\left[\frac{E_f}{\kappa_BT}+2\ln \left({e}^{-\frac{E_f}{\kappa_BT}}+1\right)\right]+\frac{ie^2}{4\pi \mathrm{\hslash}}\ln \left[\frac{2{E}_f-\left(\omega +i{\tau}^{-1}\right)\mathrm{\hslash}}{2{E}_f+\left(\omega +i{\tau}^{-1}\right)\mathrm{\hslash}}\right] $$

where *T* = 300K is the room temperature and *E*_*f*_ is the Fermi energy. Meanwhile, the intrinsic relaxation time is described as $\tau =\mu {E}_f/\mathrm{e}{\upsilon}_f^2$, where *υ*_*f*_ is the Fermi velocity and *μ* = 10000cm^2^*V*^−1^*s*^−1^ is the carrier mobility. For our proposed structure, the Fermi energies of the upper and lower graphene films are assumed as *E*_*f*1_ = 0.66*eV* and *E*_*f*2_ = 0.31*eV*, respectively.

In the simulation, we utilize the 3D FDTD method for the numerical calculation. Meanwhile, periodic boundary conditions are applied along the *x*- and *y*-directions, and perfectly matched layer is applied along the *z*-direction including both the top and bottom of the proposed device. Moreover, we utilize the non-uniform mesh to compute the simulation results, where the minimum mesh size inside the graphene layer equals 0.1 nm and gradually increases outside the graphene film to cut down on the storage space and computing time.

## Results and Discussion

**Fig. 2:**
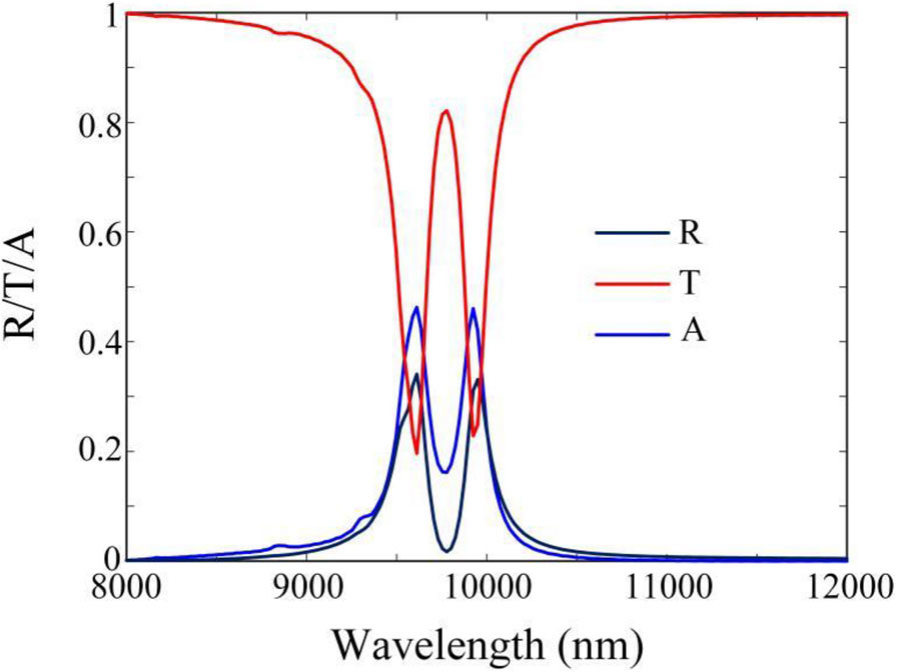
The reflection (R), transmission (T), and absorption (A) spectra of the proposed graphene-based absorber with Fermi energies *E*_*f*1_ = 0.66 eV and *E*_*f*2_ = 0.31 *eV* under the illumination of only one incident beam *I*_*1*_ in the *z* direction

Firstly, in order to clearly explain the physical mechanism, we investigate the absorption of proposed CPA under normal illumination of only one incident beam *I*_*1*_ in the *z*-direction. Since the graphene-based CPA is in the symmetry environment, the combined reflection and transmission coefficients can be expressed as *r* = *η* and *t* = 1 + *η*, respectively, where *η* is the self-consistent amplitude related to the graphene hybrid waveguide. Thus, the absorption is derived as *A* = 1 − |*r*|^2^ − |*t*|^2^ =  − 2*η*^2^ − 2*η*. The condition of maximum absorption is *∂A*/*∂η* = 0 (*∂A*^2^/*∂η*^2^ is real and negative) and we get $\eta =-\frac{1}{2}$. Then, the limit to maximum absorption is *A*_max_ = 0.5. In our simulation, when only one incident beam *I*_1_ vertically illuminates on the proposed absorber, due to the plasmonic resonances on the double graphene films, which are emerged by incident light through the silicon array for the mechanism of guided-mode resonance, then the coupling effect between the double graphene films leads to the dual-band absorption peaks, as demonstrated in Fig. 2. However, both two absorption peaks are less than 0.5, which accord with the absorption limit.

Then, when *I*_*1*_ and *I*_*2*_ vertically incident on the proposed structure from opposite sides, the schematic diagram is shown in Fig. 1a. Meanwhile, *O*_*1*_ and *O*_*2*_ can also be assumed as the intensities of emergent lights from the bottom and top of the proposed CPA. The relationship between the incident lights and emergent lights is demonstrated by the scattering matrix:

2 $$ \left[\begin{array}{c}{O}_2\\ {}{O}_1\end{array}\right]=\left[\begin{array}{cc}{r}_{11}& {t}_{12}\\ {}{t}_{21}& {r}_{22}\end{array}\right]\left[\begin{array}{c}{I}_1\\ {}{I}_2\end{array}\right] $$

When the incoherent absorption limit is satisfied (i.e., *r*_11_ = *r*_22_ =  − 0.5 and *t*_12_ = *t*_21_ = 0.5), by considering the relationship *I*_2_ = *αI*_1_ exp(*iφ* + *ikz*) with *z* = 0, the coherent absorption *A*_co_ of the proposed graphene-based CPA is express as [36]:

3 $$ {A}_{\mathrm{co}}=1-\frac{{\left|{O}_1\right|}^2+{\left|{O}_2\right|}^2}{{\left|{I}_1\right|}^2+{\left|{I}_2\right|}^2}=1-\frac{1+{\alpha}^2-2\alpha \cos \left(\varphi \right)}{2\left(1+{\alpha}^2\right)} $$

Thus, according to Eq. (3), *A*_co_ can be manipulated by changing *α* and *φ*. In particular, if *α* = 1, *A*_co_ can be tuned from the minimum *A*_co − min_ = 0 to the maximum *A*_co − max_ = 1 when *φ* varies from (2*N* + 1)*π* to 2*Nπ*.

**Fig. 3:**
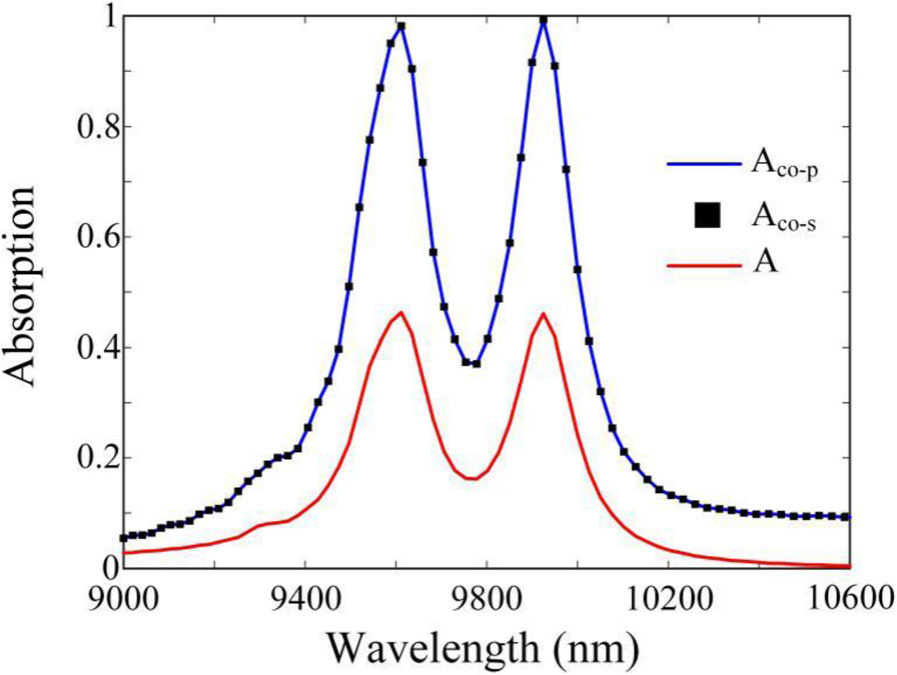
The absorption spectra of the proposed graphene-based absorber under the illumination of only one incident beam (red curve), and under coherent illumination with *p* polarization (blue curve) and s polarization (black curve)

As illustrated in Fig. 3, when two incident lights with *φ* = 0 and *α* = 1 are coherent illuminated on the proposed structure, dual-band perfect absorption peaks can be achieved in *λ*_1_ = 9611 nm and *λ*_2_ = 9924 nm, respectively. Moreover, compared with the absorption under the illumination of only one incident beam, the absorption of the proposed graphene-based CPA has been significantly enhanced. It is worth noting that due to its center symmetric feature, the proposed CPA also demonstrates polarization-insensitive. As shown in Fig. 3, whether the incident lights with *p* or *s* polarization, the absorption spectrum remains the same.

**Fig. 4:**
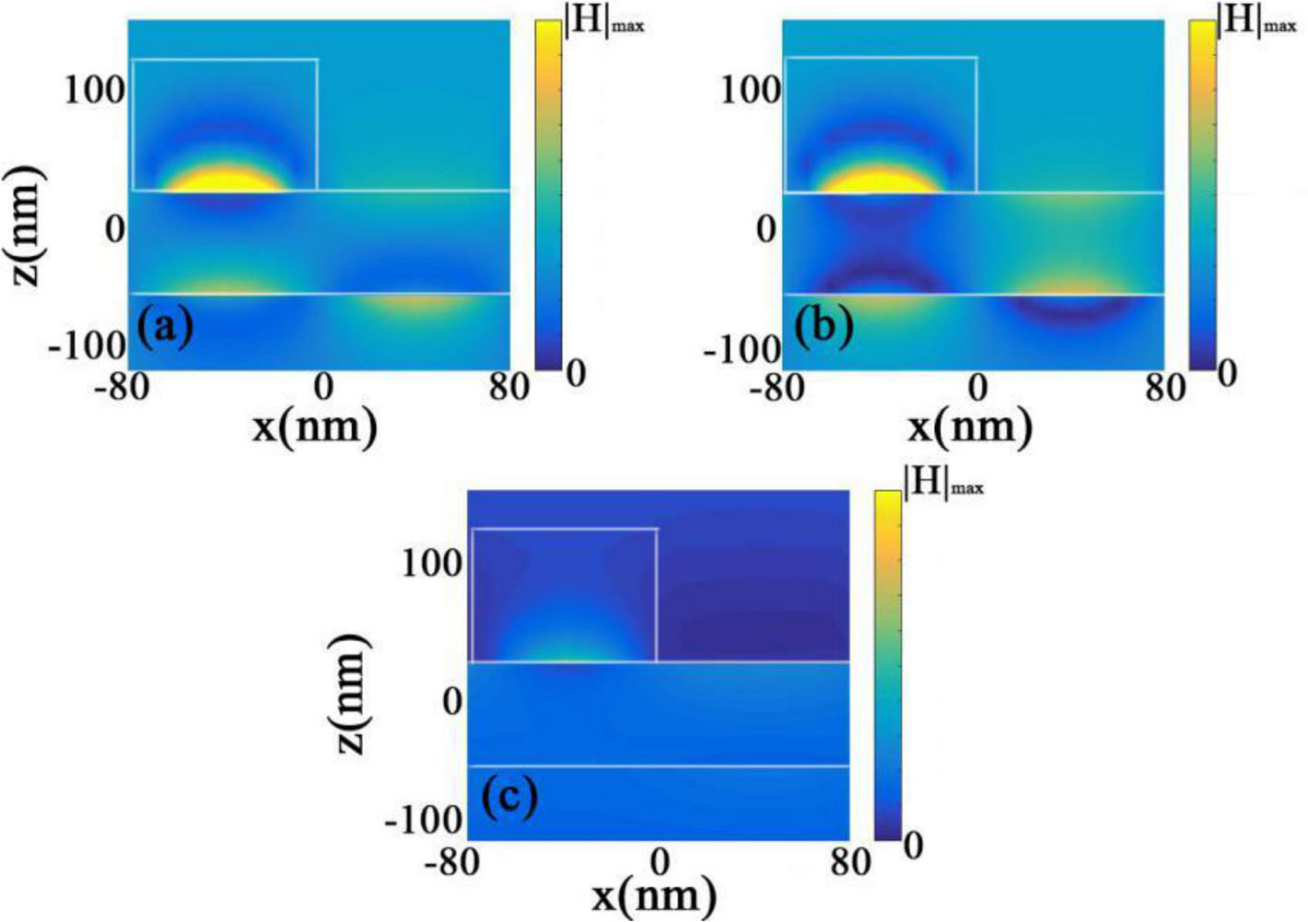
Contour profiles of normalized magnetic fields of the proposed graphene-based CPA (**a**) at λ_1_ = 9611 nm, (**b**) λ_2_ = 9924 nm, and (**c**) λ_3_ = 9000 nm

To clearly demonstrate the features of proposed CPA, we illustrate the magnetic fields around the double-layers graphene waveguide at the wavelengths of absorption peaks. As described in Fig. 4a, b, the magnetic fields around two graphene layers are both gathered and trapped at the wavelengths of absorption peaks. However, for the upper graphene film, the magnetic fields are mainly confined between the silicon squares and the upper graphene film, which correspond to the localized plasmon mode. Moreover, once another graphene film is added below the upper graphene film, light energies will transfer from the upper layer to the lower one due to the guided-mode resonance. Then, the coupling effect between the upper graphene layer and the lower one enhances the optical fields and concentrates the light energies in the proposed structure, which leads to the dual-band absorption peaks, as shown in Fig. 3. On the other hand, at the wavelength of 9000 nm, there are few strengthened optical fields surrounding two graphene films, because it is far away from resonance wavelengths, as demonstrated in Fig. 4c.

**Fig. 5:**
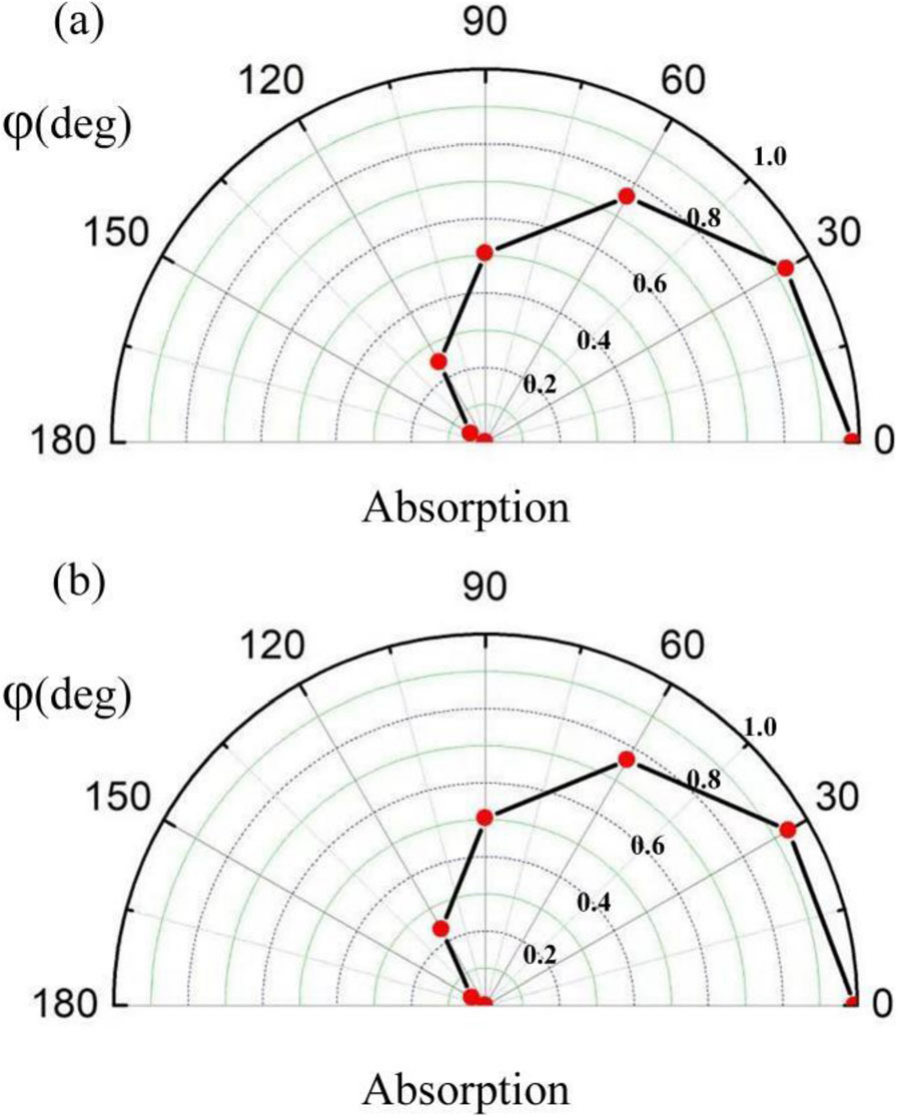
The absorption of proposed CPA with different phase difference at the peaks of **a** λ1 = 9611 nm and **b** λ2 = 9924 nm, respectively

Next, for the sake of displaying all-optical modulation characteristics, we demonstrate the coherent absorption of proposed absorber with different phase differences *φ*, as illustrated in Fig. 5. Meanwhile, the relative amplitude *α* of coherent incident lights is set as 1, and the other structural parameters are kept as the same as that in Fig. 1. As depicted in Fig. 5a, b, by increasing *φ* from 0 to π, two absorption peaks at 9611 nm and 9924 nm decrease continuously from 0.982 and 0.993 to almost 0, respectively. Thus, the modulation contrast can be as high as 34.8 dB and 35.2 dB at the two coherent absorption peaks with different *φ*, which shows a significant all-optical modulation property.

**Fig. 6:**
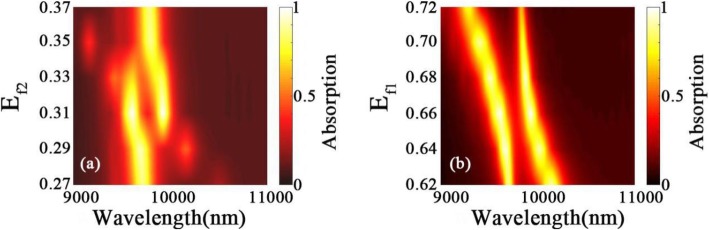
Absorption spectra as a function of the wavelength and Fermi levels of **a** lower-layer graphene and **b** upper-layer graphene. The other structural parameters are the same as Fig. 1

In the following, for our four layers (silicon array-graphene waveguide/silica layer/graphene film/silica substrate) system, combined with continuous boundary conditions and the Maxwell equations, the dispersion relation can be expressed as [37]:

4 $$ \exp \left(-2{k}_2{d}_1\right)=\frac{1+\frac{\varepsilon_2{k}_1}{\varepsilon_1{k}_2}}{1-\frac{\varepsilon_2{k}_1}{\varepsilon_1{k}_2}}\bullet \frac{\left(1+\frac{\varepsilon_2{k}_3}{\varepsilon_3{k}_2}\right)\left(1+\frac{\varepsilon_3{k}_4}{\varepsilon_4{k}_3}\right)+\left(1-\frac{\varepsilon_2{k}_3}{\varepsilon_3{k}_2}\right)\left(1-\frac{\varepsilon_3{k}_4}{\varepsilon_4{k}_3}\right)\exp \left(-2{k}_3{d}_g\right)}{\left(1-\frac{\varepsilon_2{k}_3}{\varepsilon_3{k}_2}\right)\left(1+\frac{\varepsilon_3{k}_4}{\varepsilon_4{k}_3}\right)+\left(1+\frac{\varepsilon_2{k}_3}{\varepsilon_3{k}_2}\right)\left(1-\frac{\varepsilon_3{k}_4}{\varepsilon_4{k}_3}\right)\exp \left(-2{k}_3{d}_g\right)} $$

where, *ε*_*i*_ and *k*_*i*_ (*i* = 1, 2, 3, 4) are the permittivities and wave vectors of the silicon array-graphene waveguide (*i* = 1), silica layer (*i* = 2), graphene film (*i* = 3), and silica substrate (*i* = 4), respectively. *d*_*g*_ is the thickness of graphene. Thus, by properly manipulating the Fermi energies of two graphene films, the features of plasmonic modes sustained by two graphene films could be significantly and independently controlled. As seen in Fig. 6a, b, the absorption spectra of the proposed CPA can be flexibly and separately manipulated by altering the Fermi energies of lower-layer or upper-layer graphene film. When the Fermi energy *E*_*f*1_ of upper-layer graphene remains unchanged and the Fermi energy *E*_*f*2_ of lower-layer graphene decreases from 0.31 to 0.27 eV, the absorption peak at *λ*_1_ red-shifts and keeps the value almost unchanged, while the absorption peak at *λ*_2_ reduces rapidly and even disappears under *E*_*f*2_ = 0.27 eV, as shown in Fig. 6a. On the contrary, when *E*_*f*2_ increases from 0.31 to 0.37 eV, the absorption peak at *λ*_1_ reduces rapidly and almost disappears under *E*_*f*2_ = 0.37 eV, while the absorption peak at *λ*_2_ blue-shifts and keeps the value almost unchanged. Thus, the dual-band proposed perfect absorber can be changed to narrow-band perfect absorber by separately altering the *E*_*f*2_. On the other hand, when *E*_*f*2_ remains unchanged and *E*_*f*1_ increases from 0.62 to 0.72 eV, both two absorption peaks blue-shifts and keeps their values almost unchanged over a wide wavelength range, which demonstrates a significantly tunable characteristic. Compared with the other absorbers based on the discrete graphene patterns, it is worth noting that two graphene films of the proposed CPA are in the continuous form, which is more convenient to get excellent tunability.

**Fig. 7:**
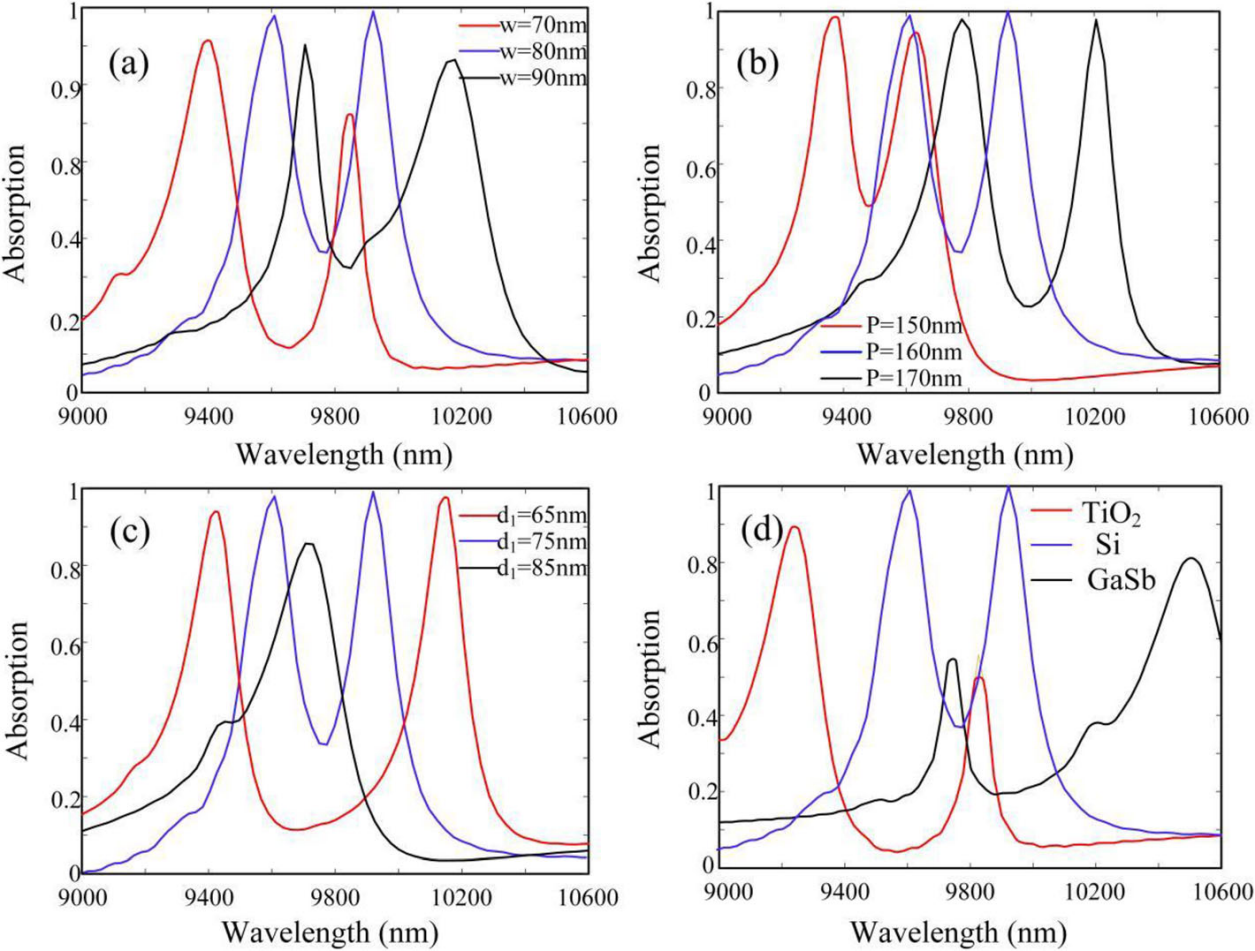
Light absorption of proposed CPA with different **a** *p*, **b** *w*, **c** *d*_1_, and **d** different dielectric array, respectively. The other parameters are the same as Fig. 2

In addition, we investigate the influences of different structure parameters on the optical absorption of proposed CPA, as shown in Fig. 7. Since each silicon square performances as a Fabry-Perot resonator for the localized plasmon mode, and the resonant wavelength is remarkably sensitive to the width of silicon squares. Thus, as shown in Fig. 7a, when the *w* is increased, dual-band absorption peaks are both red-shifted due to the increment of effective resonance wavelength of localized plasmon mode. Moreover, the filling factor will increase with *w*, which further reinforces the intensity of field enhancement and concentration between neighboring silicon square and inside graphene. Thus, the absorption efficiency will firstly increase with *w*. However, with the continuous increment of filling factor, too many areas of graphene will be covered by silicon squares. As a result, the absorption efficiency will subsequently decrease with the increment of w. Then, as shown in Fig. 7b, the absorption peaks will also be noticeably red-shifted with the increment of *p*, because the resonant wavelength of localized plasmon mode becomes larger. Furthermore, it is noted that the resonant frequency of the plasmonic mode supported by the lower-layer graphene strongly depends on the separation distance *d*_1_. As shown in Fig. 7c, when *d*_1_ is increased, the nearfield coupling strength between the upper- and lower-layer resonance modes will become more and more weak, which lead the dual-band absorption peaks eventually to degenerate into one peak. Meanwhile, we also investigate the absorption of proposed CPA with different dielectric array. As shown in Fig. 7d, the performances of dual-band CPA with whether the TiO_2_ array (*n*_*T*_ = 2.9) or the GaSb array (*n*_*G*_ = 3.8) is not better than the one with silicon array. Moreover, it is worth noting that the wavelengths of absorption peaks are red-shifted with the increment of the refractive index of the dielectric array.

## Conclusion

As mentioned before, most reported graphene-based perfect absorbers are polarization-sensitive and focused on the narrowband or broadband perfect absorbers, dual-band graphene-based perfect absorbers are seldom investigated in the mid-infrared regime. In this paper, we have designed a tunable dual-band and polarization-insensitive CPA in the mid-infrared regime, and the corresponding absorption features are discussed by using the scattering matrix and FDTD simulation, which illustrate that dual-band perfect absorption peaks are achieved in 9611 nm and 9924 nm, respectively. Moreover, due to its center symmetric feature, the proposed CPA also exhibits polarization-insensitive. Meanwhile, the coherent absorption peaks can be all-optically modulated by altering the relative phase between two reverse incident lights. Furthermore, by manipulating the Fermi energies of two graphene layers, two coherent absorption peaks can move over a wide spectrum range, and our designed CPA can also be changed from dual-band CPA to narrowband CPA. On the other hand, for the proposed CPA, subwavelength metamaterials based on silicon squares can be integrated for the current CMOS technology, and chemical vapor deposition (CVD) grown graphene can be transferred over the silica layer using standard transfer techniques [38]. Moreover, compared with the devices based on patterned graphene, our structure keeps graphene in the continuous form, which has the benefit of preserving the high mobility of graphene and simplifies the fabrication processes as well as the doping configuration. In recent years, some research groups have tried to design some graphene-based devices in an experiment based on the above methods [39–41]. Therefore, we believe it is possible to fabricate our proposed structure with similar processing, and our proposed graphene-based CPA can find some potential applications in the field of developing nanophotonic devices at the mid-infrared regime.

## Data Availability

All data generated or analyzed during this study are included in this published article.

## Abbreviations

2D: Two-dimension
CPA: Coherent perfect absorber
FDTD: Finite-difference time-domain
ITO: Indium tin oxide
SPPs: Surface plasmon polaritons
TMDCs: Transition-metal dichalcogenides
